# Supplementary material for: The Effect of Atm Loss on Radiosensitivity of a Primary Mouse Model of Pten-Deleted Brainstem Glioma
Source: Cancers (Basel). 2022 Sep 17;14(18):4506. doi: 10.3390/cancers14184506 (PMC9496888; doi:10.3390/cancers14184506)
Supplement: Supplementary file 1 [file cancers-14-04506-s001.zip › cancers-1883869-supplementary.pdf]

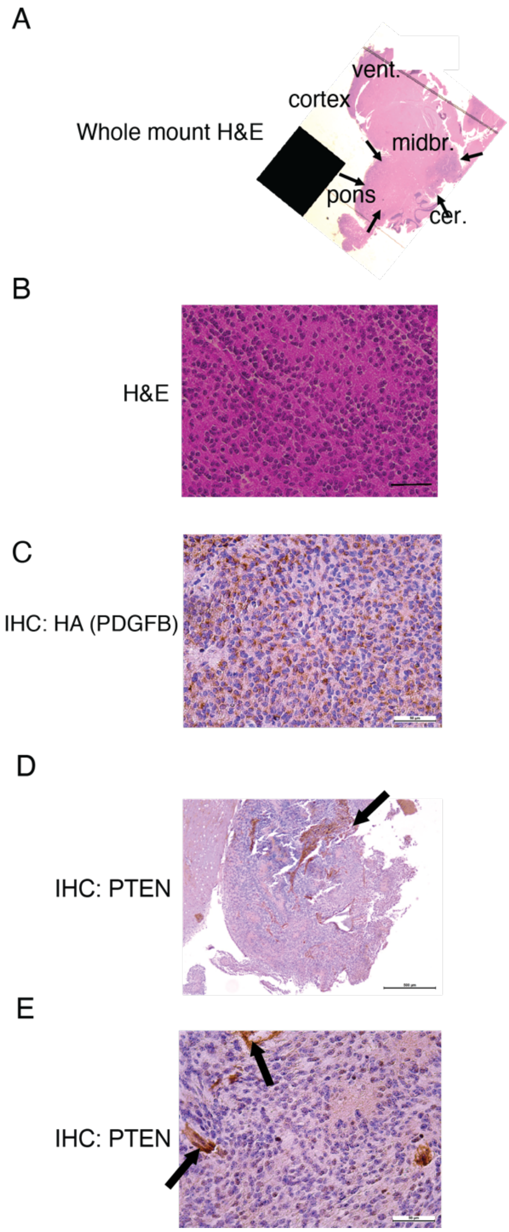

Supplementary Figure S1. Characterization of nPtenA$^{FL/+}$ brainstem gliomas.
(A) Whole-mount H&E slide showing expansile tumors in the brainstem of nPtenA$^{FL/+}$ mice. Midbr., midbrain; vent, lateral ventricle; cer., cerebellum.
(B) Magnified H&E slides for tumors from nPtenA$^{FL/+}$ mice. Scale bar represents 50 μm.
(C) Immunohistochemistry staining for HA-tagged PDGFB in tumors from nPtenA$^{FL/+}$ mice. Scale bar represents 50 μm.
(D) 5X mount showing immunohistochemistry for PTEN of tumors centered in the brainstem (pons) with lack of PTEN reactivity, and diffuse infiltrating borders (arrows) for tumors from nPtenA$^{FL/+}$ mice. Scale bar represents 500 μm.
(E) 40X mount shows complete loss of PTEN in tumor cells but not normal blood vessels (arrows) in nPtenA$^{FL/+}$ mice. Scale bar represents 50 μm.
